# The relationship between autistic camouflaging and mental health: a scoping review

**DOI:** 10.3389/fpsyt.2026.1701615

**Published:** 2026-06-02

**Authors:** Ellie Kiger Hodge, Katherine Kuhl Meltzoff

**Affiliations:** 1 University of California, Riverside, CA, United States; 2 School of Education, University of California, Riverside, CA, United States

**Keywords:** ASC, ASD, camouflaging, masking, mental health

## Abstract

**Background:**

Autistic camouflaging, also referred to as “masking” or “adaptive morphing”, refers to the conscious and unconscious use of strategies to socially fit in, mimic neurotypical behavior, or suppress or “mask” ASD traits. Some studies suggested a relationship between camouflaging and poor mental health.

**Objective:**

The goal of this review is to answer the question: “What is the current state of literature regarding the relation between autistic camouflaging and mental health?”

**Methods:**

We reviewed qualitative, quantitative, and mixed-method peer-reviewed publications regarding autistic camouflaging and mental health from four databases (Google Scholar, Psychinfo, PubMed, and JSTOR). Studies needed to involve autistic participants, be an experimental or observational study, and be in English. Results of said studies relating to camouflaging and depression, anxiety, stress, mental wellbeing, or burnout were summarized with effect sizes being reported when available. Data were charted using an adapted and modified Cochrane review checklist.

**Results:**

Forty-eight studies were selected based on eligibility criteria. Results indicated a positive relationship between camouflaging and poor mental health with effect sizes ranging from small to large. Mental health issues appeared to relate more with assimilation (attempting to fit in during social settings) than with compensation (mimicking and practiced skills) or masking (actively attempting to suppress ASD traits). Results for qualitative studies indicated that the relationship between camouflaging and mental health appeared to be bidirectional. Non-autistic individuals had similar relationships between camouflaging and mental health as autistic participants.

**Conclusions:**

Given the finding that camouflaging is associated with poor mental health outcomes, and qualitative findings that it may contribute to poor mental health, caution should be taken regarding the encouraging of camouflaging behaviors, especially assimilation behaviors, by interventions. Future research is needed into these relationships, especially as said relationship was present in non-autistic samples as well.

## Introduction

Autism spectrum disorder (ASD) is a neurodevelopmental disorder with a prevalence of 1 in 100 children worldwide and 1 in 31 in the United States (1, 2). Although diverse in its presentation, key traits include social-communicative challenges and the presence of restricted interests and repetitive behaviors. Autism has a high level of co-occurrence with other psychiatric disorders and mental health conditions, with social anxiety and attention-deficit/hyperactivity disorder having the highest rates of co-occurrence (3). Additionally, autistic individuals have higher rates of suicidal ideation and attempts compared to their neurotypical peers (4, 5). Autistic children have reported increased feelings of loneliness compared to neurotypical (NT) peers and also struggle more to define said experiences of loneliness (6). While the exact reasons for these high levels of mental health issues may vary, one possible contributing factor is autistic camouflaging.

Autistic camouflaging, also referred to as “masking” or “adaptive morphing”, is a recently studied phenomenon and refers to the conscious and unconscious use of strategies to socially fit in, mimic neurotypical behavior, or suppress or “mask” ASD traits (7, 8). Definitions frequently describe camouflaging as a behavior action used by autistic individuals to live in a majority non-autistic society (7, 9, 10). Frequently, definitions specify that these strategies and actions are used to hide autistic traits and appear more neurotypical (8, 9). Note, however, that due to the novel nature of this topic, there is not yet a universal definition of camouflaging and the exact definition varies between researchers (10). The current review defines camouflaging as the set of conscious or unconscious behaviors and strategies used to mimic neurotypical behaviors and hide autistic traits or to better fit in a majority neurotypical society. There are two primary ways to measure camouflaging. First is the discrepancy model of measurement which quantifies camouflaging as the discrepancy between internal (self-perceived) autism severity and externally (clinician-perceived) autism severity as measured by the Autism Diagnostic Observation Schedule (ADOS; 11) or measures of social intelligence and external autism severity (12). Secondly there is the Camouflaging Autistic Traits Questionnaire (CAT-Q), developed in 2019, which is the first and as of this writing, only, validated self-report measure of camouflaging traits for autistic individuals (13). Crucially, development of the CAT-Q was informed by the lived experiences of autistic individuals. Factor analysis revealed three main subcomponents to the questionnaire: compensation, masking, and assimilation. Compensation includes questions that ask about mimicking others and media as well as preparing social skills in advance of social interactions. Masking involves body language monitoring and adjustment as well as awareness of one’s impression. Assimilation relates to feelings of authenticity or freedom to be authentic as well as questions regarding avoiding, forcing social interaction, social support and conversation flow. The CAT-Q is limited as it has only been validated for ages 16 and older and self-report measures may be biased. While a parent-report version of the CAT-Q has been developed, validation of this version is lacking as compared to the self-report version.

Anecdotally, many autistic individuals report camouflaging as taxing and report negative effects on their mental and emotional health. To our knowledge, only three reviews have been done with a primary focus on the relationship between camouflaging and mental health. A systematic review found that camouflaging led to negative psychosocial outcomes, and although it addressed mental health peripherally, the focus was on psychosocial factors (e.g. stigma and loneliness; 14). One review and meta analysis found that camouflaging was related to negative mental health outcomes, but did not include qualitative studies, and therefore was missing insight from autistic perspectives (15). One review of 24 papers had overall consequences of camouflaging as the primary focus and found that negative mental health outcomes were a potential impact of camouflaging (16). Other reviews with broader scopes have explored camouflaging as well and in doing so explored the relationship between camouflaging and mental health as well as other related factors. (7, 9, 17). The current review includes qualitative, quantitative, and mixed-method studies to answer the question, “What is the current state of literature regarding the relation between autistic camouflaging and mental health?”. Considering the lack of unified definition or measurement of camouflaging and the breadth of methodologies used in camouflaging and mental health studies (i.e., qualitative, quantitative, varying mental health measures), a scoping review was chosen over a systematic review. Additionally, given how relatively new this area of study is, a scoping review was identified as more appropriate for capturing the overall scope of the field regarding this relationship rather than a systematic review attempting to answer a more specific question.

## Methods

This scoping review followed a methodological framework adapted from Arksey and O’Malley (18) in addition to following the PRISMA extension for scoping reviews checklist (19). This review was not preregistered in any online database (eg. PROSPERO). Google Scholar, PsychInfo, JSTOR, and PubMed were searched for this review. Google Scholar served to collect a wider net of sources while PubMed and PsychInfo served to ensure relevant sources were found. JSTOR provided a non-medical database to further widen the search net. Searches for PsychInfo and Google Scholar were completed February 3rd-5th of 2025. Additional searches on December 29th (Google Scholar) and 31st (PsychInfo) of 2025 were performed to update for publications up to July 22nd, 2025. JSTOR was searched February 1st, 2026 and PubMed was searched March 18th, 2026. Search phrases are organized by database and constructs in **Table 1**. Search phrases included “allintitle: autistic camouflaging OR masking OR “adaptive morphing” -review”, “allintitle: autism camouflaging OR masking OR “adaptive morphing” -review”, “allintitle: autism camouflage OR mask OR “adaptive morph” -review”, and “allintitle: autistic camouflage OR mask OR “adaptive morph” -review” for Google Scholar. Search phrases included “Title: autistic AND Title: mask AND Peer-Reviewed Journals only”, “Title: autistic AND Title: masking AND Peer-Reviewed Journals only”, “Title: autism AND Title: masking AND Peer-Reviewed Journals only”, “Title: autism AND Title: mask AND Peer-Reviewed Journals only”, “Title: autism AND Title: adaptive morphing AND Peer-Reviewed Journals only”, “Title: autistic AND Title: adaptive morphing AND Peer-Reviewed Journals only”, “Title: autism AND Title: adaptive morph AND Peer-Reviewed Journals only”, “Title: autistic AND Title: adaptive morph AND Peer-Reviewed Journals only”, “Title: autism AND Title: camouflage AND Peer-Reviewed Journals only”, “Title: autistic AND Title: camouflage AND Peer-Reviewed Journals only”, “Title: autistic AND Title: camouflaging AND Peer-Reviewed Journals only”, “Title: autism AND Title: camouflaging AND Peer-Reviewed Journals only” for PsychINFO. For JSTOR, search terms included “((ti:”autism”) AND (ti:”camouflage”))”, “((ti:”autism”) AND (ti:”mask”))”, “((ti:”autism”) AND (ti:”adaptive morph”))”, “((ti:”autistic”) AND (ti:”camouflage”))”, “((ti:”autistic”) AND (ti:”mask”))”, “((ti:”autistic”) AND (ti:”adaptive morph”))”, “((ti:”autism”) AND (ti:”camouflaging”))”, “((ti:”autism”) AND (ti:”masking”))”, “((ti:”autism”) AND (ti:”adaptive morphing”))”, “((ti:”autistic”) AND (ti:”camouflaging”))”, “((ti:”autistic”) AND (ti:”masking”))”, and “((ti:”autistic”) AND (ti:”adaptive morphing”))”. For PubMed, search terms included “(mask[Title]) AND (autism[Title]), “(masking[Title]) AND (autism[Title]), “(mask[Title]) AND (autistic[Title])”, (masking[Title]) AND (autistic[Title])”, “(camouflage[Title]) AND (autism[Title])”, “(camouflaging[Title]) AND (autism[Title])”, “(camouflage[Title]) AND (autistic[Title])”, “(camouflaging[Title]) AND (autistic[Title])”, “(adaptive morph[Title]) AND (autism[Title])”, “(adaptive morphing[Title]) AND (autism[Title])”, “(adaptive morph[Title]) AND (autistic[Title])”, “(adaptive morphing[Title]) AND (autistic[Title])”. All such articles pulled aside this way had their abstract searched for references to mental health factors (i.e., mental health, depression, stress, exhaustion, anxiety, suicidality, etc). All studies that possessed one of these terms in the abstract were pulled for consideration for inclusion in this review. Factors that were adjacently related to mental health (neuroticism, stigma, positive affect, identity, etc) were not considered for study selection. After initial study selection, the references of each study were examined for any articles with titles featuring reference to “depression”, “anxiety”, “mental wellbeing”, “suicidality”, “stress”, “emotion”, “exhaustion”, “burn-out”, “self-esteem”, or variants of said terms (i.e., “anxious”).

**Table 1:** Search terms by construct and database.

Databases Searched	Google Scholar	PsychInfo	JSTOR	PubMed
Autism + Mask	allintitle: autism camouflage OR mask OR “adaptive morph” -review	Title: autism AND Title: mask AND Peer-Reviewed Journals only	((ti:”autism”) AND (ti:”mask”))	(mask[Title]) AND (autism[Title])
Autism + Masking	allintitle: autism camouflaging OR masking OR “adaptive morphing” -review	Title: autism AND Title: masking AND Peer-Reviewed Journals only	((ti:”autism”) AND (ti:”masking”))	(masking[Title]) AND (autism[Title])
Autistic + Mask	allintitle: autistic camouflage OR mask OR “adaptive morph” -review	Title: autistic AND Title: mask AND Peer-Reviewed Journals only	((ti:”autistic”) AND (ti:”mask”))	(mask[Title]) AND (autistic[Title])
Autistic + Masking	allintitle: autistic camouflaging OR masking OR “adaptive morphing” -review	Title: autistic AND Title: masking AND Peer-Reviewed Journals only	((ti:”autistic”) AND (ti:”masking”))	(masking[Title]) AND (autistic[Title])
Autism + Camouflage	allintitle: autism camouflage OR mask OR “adaptive morph” -review	Title: autism AND Title: camouflage AND Peer-Reviewed Journals only	((ti:”autism”) AND (ti:”camouflage”))	(camouflage[Title]) AND (autism[Title])
Autism + Camouflaging	allintitle: autism camouflaging OR masking OR “adaptive morphing” -review	Title: autism AND Title: camouflaging AND Peer-Reviewed Journals only	((ti:”autism”) AND (ti:”camouflaging”))	(camouflaging[Title]) AND (autism[Title])
Autistic + Camouflage	allintitle: autistic camouflage OR mask OR “adaptive morph” -review	Title: autistic AND Title: camouflage AND Peer-Reviewed Journals only	((ti:”autistic”) AND (ti:”camouflage”))	(camouflage[Title]) AND (autistic[Title])
Autistic + Camouflaging	allintitle: autistic camouflaging OR masking OR “adaptive morphing” -review	Title: autistic AND Title: camouflaging AND Peer-Reviewed Journals only	((ti:”autistic”) AND (ti:”camouflaging”))	(camouflaging[Title]) AND (autistic[Title])
Autism + Adaptive Morph	allintitle: autism camouflage OR mask OR “adaptive morph” -review	Title: autism AND Title: adaptive morph AND Peer-Reviewed Journals only	((ti:”autism”) AND (ti:”adaptive morph”))	(adaptive morph[Title]) AND (autism[Title])
Autism + Adaptive Morphing	allintitle: autism camouflaging OR masking OR “adaptive morphing” -review	Title: autism AND Title: adaptive morphing AND Peer-Reviewed Journals only	((ti:”autism”) AND (ti:”adaptive morphing”))	(adaptive morphing[Title]) AND (autism[Title])
Autistic + Adaptive Morph	allintitle: autistic camouflage OR mask OR “adaptive morph” -review	Title: autistic AND Title: adaptive morph AND Peer-Reviewed Journals only	((ti:”autistic”) AND (ti:”adaptive morph”))	(adaptive morph[Title]) AND (autistic[Title])
Autistic + Adaptive Morphing	allintitle: autistic camouflaging OR masking OR “adaptive morphing” -review	Title: autistic AND Title: adaptive morphing AND Peer-Reviewed Journals only	((ti:”autistic”) AND (ti:”adaptive morphing”))	(adaptive morphing[Title]) AND (autistic[Title])

Six-hundred and eighty-six results were obtained during the search using the above criteria. Each of the 686 results identified by the search had their abstracts examined for eligibility. Sources lacking abstracts were skimmed briefly to confirm their status as peer-reviewed and published studies. Fifty-five of these had their full papers pulled to be read and analyzed for eligibility. Both abstracts and full texts were evaluated for eligibility based on the following criteria:

1. Report on social camouflaging (also known as “masking” or “adaptive morphing”).
2. Explore the direct relation between camouflaging and mental health.
3. Be published in a peer-reviewed journal.
4. Involve autistic participants. Examinations solely of media were not included.
5. Be an experimental or observational study and not a review, editorial, conference poster, conference presentation.
6. Have available English translations.
7. Feature results in an autistic sample or sub-sample.

In order to ensure an adequate number of studies related to specific mental health concepts, after initial collection of sources from the first searched databases, we narrowed it down to the most common mental health concepts studied as potentially related to camouflaging in the initially pulled quantitative studies. This was done to improve interpretability and readability of synthesized findings. Burnout was also chosen due to recent literature suggesting camouflaging is a key contributor to autistic burnout (20, 21). Based on these criteria, 56 studies were pulled and 34 were selected. Eight more studies were obtained from the references of the initial 34 studies. After initial submission, the authors searched for articles dated between February, 2025 and September 22nd, 2025 and found six additional articles using the above criteria. This provided a total of forty-eight studies included in this review.

Data was charted using an adapted and modified 2011 Cochrane data extraction checklist (22). The data charting template was edited over time to include clarifications to correct any confusion from research assistant coders. The primary author and six research assistants extracted data separately from the papers’ primary report. Studies’ supplementary information and data were examined by the primary author. Participant data regarding diagnosis, nationality/geographics, gender/sex and age range are reported in **Supplementary Table 1**. Statistics are reported for each study with quantitative results about the relationship between camouflaging and mental health in **Supplementary Tables S3**–**S8**. When available, coefficients that measure effect size (e.g. Pearson’s *r*; regression beta coefficient) are reported. Results are synthesized narratively by mental health factor and original analysis type (i.e., quantitative or qualitative).

## Results

**Figure 1:**
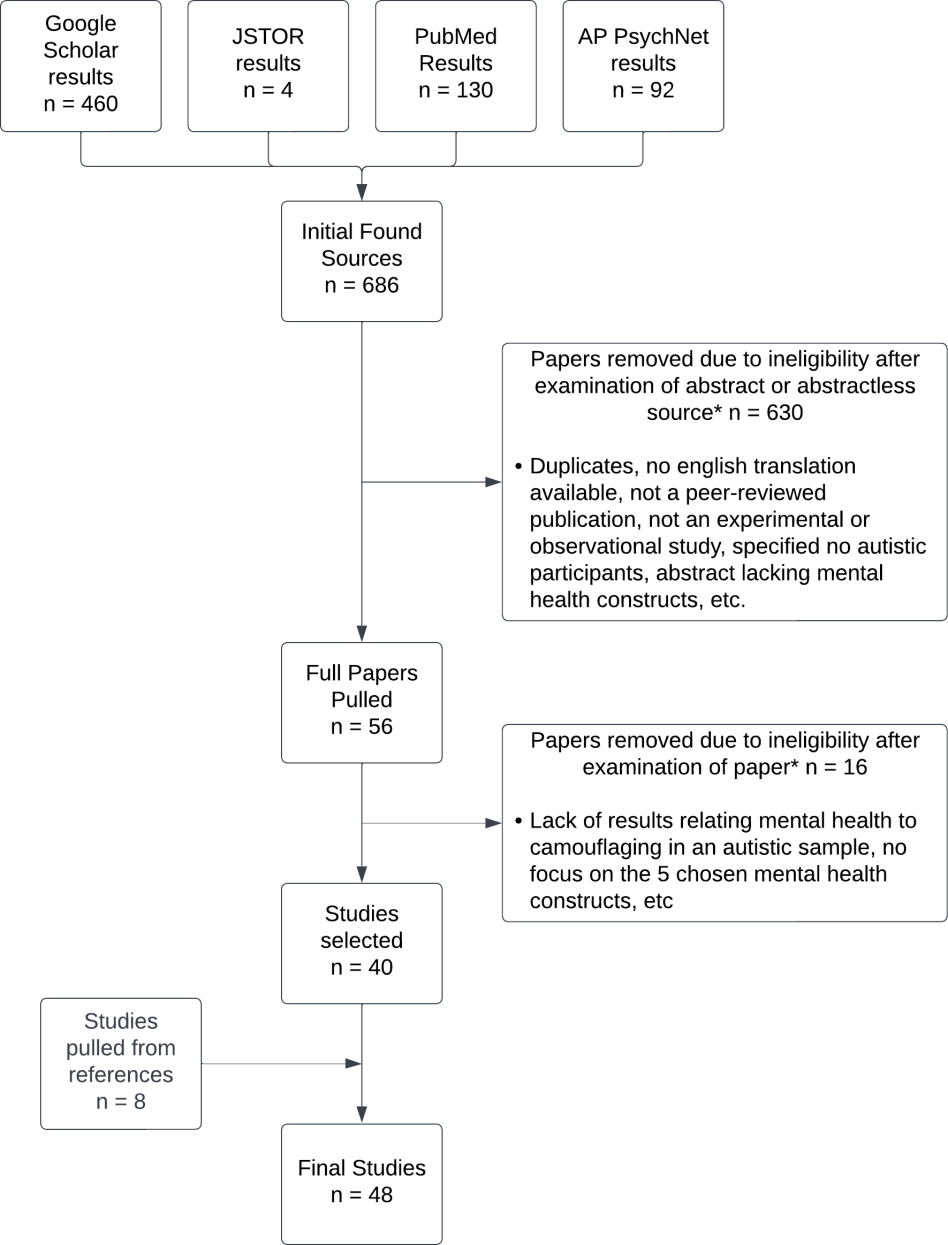
PRISMA Flowchart of Study Selection.

Of the search results, 40 papers were selected via the above initial criterion and process. Eight additional papers from the references of the initial 33 were included for an initial total of 48 papers. Of the final 48 papers, over 80% were published after 2020 and 100% were published in the past 10 years. Thus, this body of literature is still young, but interest in camouflaging seems to be increasing. Papers included studies with quantitative (n=30), qualitative (n = 13) and mixed methods (n = 5) results regarding camouflaging and mental health relations in autistic people. **Figure 1** displays a flowchart of the data selection process. Two mixed methods studies’ qualitative results did not touch on camouflaging’s relationship with mental health, as such only the quantitative results from said studies are considered here (23, 24). Similarly, on mixed methods study’s quantitative results did not examine camouflaging’s relationship with mental health and so only its qualitative results are considered here (25). Eight studies included non-autistic participants (13, 26–32). Two of these studies had groups lacking in any diagnosable developmental, neurological, or mental health conditions which could classify them as “neuroptypical”. (28, 33).

**Supplementary Table 2** displays information related to the measures used to collect mental health information as well as how camouflaging was measured for quantitative studies. The most common mental health variables collected were depression (n = 24), unspecified anxiety (e.g. anxiety in general rather than a specific anxiety disorder) and generalized anxiety (n = 21), social anxiety (n = 9), stress (n = 9), mental wellbeing (n = 9), and burnout (n = 4). The most common measure of camouflaging was the CAT-Q, including translations and adapted variants (n = 28). Seven studies used measures of camouflaging unrelated to the CAT-Q (**Supplementary Table 2**). Thirty-one of the 48 studies explicitly mentioned funding sources for their study’s research, authorship, or publication. Nine mentioned having no funding to report or reported having no funding. Two studies only specified funding of individual authors involved in said studies. The remaining six studies did not explicitly state the funding or lack there-of obtained for said studies.

### Quantitative results

Quantitative studies featured an array of methodologies to analyze camouflaging’s relationship with mental health, primarily, correlations, regressions, mediation analyses, and ANOVA’s. Correlations broadly fell into three groups: Pearson’s, Spearman’s, and unspecified. Following Cohen’s recommendations, Pearson’s correlation coefficients between.10 and.30 were labeled as small effect size, those between.30 and.50 as medium effect size, and those.50 or greater as large effect size (34, 35). Non-specified correlations were interpreted according to these effect size standards as well. Effect size of Spearman’s rho was interpreted as.20 to.40 being small,.40 to.60 being medium and.60 and greater being larger (36). For regressions, standardized beta coefficients were interpreted with small effect size being less than.2, medium being.2 to 5, and.5 and over being large (36, 37).

Regressions included linear, multiple, hierarchical, and combinations of these. For hierarchical regressions, when variance information was provided, the model with best variance explanation was reported. Otherwise, the final model was reported unless otherwise stated. Frequently, multiple covariates were present as predictors, however, for ease of data consumption, these were not reported but can be found in the original text. Unstandardized regression coefficients were converted to standardized regression coefficients for the tables. When necessary, standard deviations of subsamples were combined using https://www.statstodo.com/CombineMeansSDs.php (38). **Supplementary Tables 3**, **S4** report regression effect sizes of the relationship between camouflaging factors and mental health constructs in autistic samples. Correlations between camouflaging factors and mental health factors in non-autistic samples can be found on **Supplementary Table 5** and **Supplementary Table S6**. Regression beta coefficients for the autistic samples can be found on **Supplementary Tables S7** and **Supplementary Table S8**. Partial eta squared for ANOVA and MANCOVA are reported in the text.

#### Depression

Nineteen of the twenty-four papers to examine the relationship between camouflaging and depression in an ASD sample found significant positive relationships between the two such that greater camouflaging was associated with greater depression (12, 13, 15, 27–29, 39–51). Among these, papers reporting effect sizes found these relationships ranged from small to large with most being small or medium. In one study, when controlling for age and sex, the relationship between camouflaging and depression was strong. One paper found that change in camouflaging over time was not predicted by baseline depression scores, and neither was change in depression score over time predicted by baseline camouflaging score (50). The same result was found for masking, assimilation, and compensation as replacements for camouflaging (50). For autistic samples, subscales of camouflaging (assimilation, compensation, masking) were examined in five papers (13, 28, 29, 50, 52). Depression had a stronger relationship with assimilation than with camouflaging, compensation, or masking in four of these papers (13, 28, 29, 52). In one study, depression had a stronger relationship with assimilation than total camouflaging when camouflaging scores and subscores predicted anxiety but not when anxiety predicted camouflaging and camouflaging subscores (50). For autistic samples, compensation did not relate to depression in three papers (29, 52), while masking did not relate to depression in four papers (28). In three studies a non-autistic sample was included and analyzed (27–29). Results from the non-autistic groups indicated that depression had a moderate to strong positive relationship with camouflaging. Interestingly, this relationship was descriptively larger for the non-autistic sample than for the autistic samples (27–29). In one paper, a CAT-Q score of 125 had the highest increase in probability of scoring above depression diagnostic cut-off on the Patient Health Questionnaire (PHQ) compared to other CAT-Q scores (45).

Some studies descriptively suggested that gender or sex may influence the relationship between camouflaging and depression with one study finding no significant relationship between camouflaging and depression in women (12, 43). Other studies suggested that gender does not affect said relationship (45, 46, 48, 53). One study suggested that the relationship between camouflaging and depression may have cross-national variation with the relationship better being explained by a linear model in a UK study sample and a quadratic model in a Japanese sample (48). However a multi-cultural study featuring a sample from eight nations found no influence of nationality on the relationship between camouflaging and depression (46). Four of the twenty-four papers failed to find any relationship in any of the reported analyses (24, 26, 53, 54).

Overall, twenty of twenty-four studies showed that there is a positive relationship such that higher camouflaging or higher camouflaging subscales were associated with greater depression. Strength of this relationship varied heavily by study, but most effect sizes were weak to moderate. For camouflaging subscales, assimilation had a stronger relationship with depression than compensation or masking. In papers that included analyses from both non-autistic and autistic individuals, the relationship between camouflaging and depression was numerically larger for the non-autistic sample.

#### Anxiety

Seventeen studies examined camouflaging and anxiety in general in an autistic sample and found significant results; reported effect sizes ranged from small to large (23, 24, ηp² = .034; 13, 27–29, 41–51). One study found that in a hierarchical regression model, a quadratic model of camouflaging was determined to have better fit over a linear model of camouflaging (48). Additionally, when controlling for age and sex, there was a strong, positive relationship between anxiety and camouflaging (42). Initial findings in one investigation showed anxiety did not predict changes in camouflaging over time, and baseline camouflaging did not predict changes in anxiety over time (50). Five studies examined the subscales of camouflaging and how they relate to anxiety (13, 28, 29, 50, 52). For autistic groups, anxiety had a stronger relationship with assimilation than with masking, compensation, or total camouflaging score in four studies (13, 28, 29, 52). Anxiety’s relationship with total camouflaging score was stronger than anxiety’s relationships with the compensation and masking subcomponents in three studies (13, 28, 29). In one study, anxiety had a stronger relationship with assimilation than total camouflaging when camouflaging scores and subscores predicted anxiety but not when anxiety predicted camouflaging and camouflaging subscores (50). When measuring these constructs at two timepoints, time 1 camouflaging subscale scores did not predict change in anxiety over time; neither did time 1 anxiety score predict change in any camouflaging subscale over time (50). In the three studies where a non-autistic sample was separately analyzed, anxiety had a moderate to strong positive relationship with camouflaging, and this relationship was descriptively stronger than in the autistic samples (27–29). The sub-scale findings were similar in the non-autistic samples as compared to the autistic sample except in one case where descriptively the relationships between anxiety and total camouflaging and anxiety and assimilation were the same (28). Interpretations of the sub-scale and non-autistic results are limited by the small number of studies with non-autistic subsamples or analyses with camouflaging subscales. In one paper, a CAT-Q score of 125 had the highest increase in probability of scoring above anxiety diagnostic cut-off on the Generalized Anxiety Disorder 7-item scale (GAD-7) compared to other CAT-Q scores (45).

Most studies found no influence of gender or sex on this relationship (45, 46, 48, 53). One found descriptively different relationships based on gender (43). As for influence of country, one study found no difference in the relationship based on participant nationality while another found that the relationship seemed to be best modeled quadratically in a Japanese but not in a non-Japanese sample (46, 48). Three of the 21 studies failed to find any significant quantitative relationship between camouflaging and anxiety across all of their reported analyses (12, 40, 53).

Eighteen of the twenty-one studies to examine anxiety and camouflaging or its subscales found significant relationships between the two with camouflaging increasing as anxiety increases. These relationships were numerically stronger in non-autistic participants in the two studies that included both non-autistic and autistic participants. Neither study directly measured whether the numeric difference in relationship strength between genders was statistically significant. The range of effect sizes reported in the 16 studies varied greatly.

##### Social anxiety

Eight studies examined the relationship between camouflaging and social anxiety in an autistic sample and found significant results such that as camouflaging increased so did social anxiety (13, 29, 32, 42, 45, 48, 49, 55). This relationship was significant with small to large effect sizes (when effect size was available). Three studies explored the relationship between social anxiety and camouflaging subscales (13, 29, 55). Assimilation had a moderate to strong relationship with social anxiety (13, 29, 55). Neither compensation nor masking had a significant relationship with social anxiety in one study (29). In the other studies, compensation and masking had weak to moderate correlations with social anxiety (13, 55). Controlling for autistic social traits using subscales of the Broader Autism Phenotype Questionnaire (BAP-Q; 56) increased effect size for masking but decreased effect size for camouflaging, assimilation, and compensation (55). In the two studies that examined social anxiety’s relationship with camouflaging in a non-autistic sample, effect sizes ranged from small to large (13, 29). In the two studies that examined the subscales in a non-autistic sample, effect sizes ranged from small to large with assimilation having the strongest effect sizes (13, 29).

One study examined the relationship between social anxiety diagnosis and autistic diagnosis with camouflaging, compensation, masking, and assimilation (32). Network plots showed that for the ASD and social anxiety groups, assimilation was connected with social avoidance. In the socially anxious and dual diagnosis groups, assimilation was connected with social anxiety (32). This study also found that using partial least squares structural equation modeling in a combined sample of ASD and dual diagnosis, social avoidance predicted camouflaging and masking, and social anxiety predicted assimilation (32).

In one study’s factor analysis, camouflaging and social anxiety items mostly loaded onto different factors suggesting a distinction between the constructs, however one masking item loaded onto a factor otherwise containing only social anxiety items and three social anxiety items loaded onto a factor containing eight assimilation items (55). A score of 75 on the CAT-Q had the highest increase in probability of scoring above the Liebowitz Social Anxiety Scale for Adults (LSAS) cut-off compared to other CAT-Q scores (45).

Eight of nine studies that examined social anxiety’s relationship with camouflaging found a significant relationship. One study failed to find a relationship between camouflaging and social phobia in either gender (57). The pattern of assimilation having a stronger relationship with mental health than compensation and masking was observed in some studies, but not all (13, 29, 32, 55). The pattern of non-autistic subsamples having descriptively larger effect sizes for the relationship of interest was upheld in one study (13), but not observed in all studies (29). Of the two studies that investigated the effect of sex/gender on the relationship between camouflaging and social anxiety, none found significant effects (45, 48).

Eight studies suggested that there is a positive relationship between camouflaging and social anxiety for both autistic and non-autistic samples. Gender did not have an effect on this relationship. Results did not fully support the pattern of assimilation having the strongest relationship with mental health factors. Notably, the factor analysis results call into question how well assimilation is related exclusively to camouflaging rather than other constructs.

#### Stress

Eight of nine studies that examined the relationship between camouflaging and stress in an autistic sample found significant results (24, ηp² = .054; 30, 43, 44, 46, 47, 51, 58). Camouflaging related positively to stress with small to large effect sizes such that higher camouflaging was associated with higher stress (30, 43, 46, 47, 51, 58). One study found this significant relationship when testing masking both within-subjects (e.g. variability in masking behaviors day to day within individuals) and between-subjects (e.g. differences in masking between individuals) (58). In one study, stress was related with assimilation more than for the compensation-masking subscale (30). This study also re-ran these analyses using a caregiver-report version of the camouflaging measure. Although the correlations remained significant, they had smaller effect sizes, except for autistic compensation-masking which was larger (30). Camouflaging frequency and context predicted stress with those who camouflage infrequently across contexts having lower stress than those who camouflage frequently across contexts or switch frequency based on context (np^2^ = .05; 24).

One study examined stress and camouflaging in a non-autistic sample (30). As with depression, anxiety, and mental wellbeing, relationships for stress and self-reported camouflaging and its subscales were descriptively greater for the non-autistic group than the autistic group of the same study (30). However, for caregiver-reported camouflaging, this relationship was not present (30). This is limited by only one study featuring both groups and both measurement types. Similar to anxiety and depression, assimilation had stronger relationships with stress than total camouflaging score, which in turn was stronger than the other subscale(s), however only one study examined these subscales (30). There were mixed results regarding gender’s influence on the relationship between camouflaging and stress. One study’s descriptive results suggested that camouflaging’s relationship with stress differed between genders (43) In two others, results suggested that sex or gender did not affect camouflaging’s relationship with stress (46, 58). When measuring the effects of masking on stress four hours later, one study found that while those who reported masking more on average each day reported more stress four hours later, individually masking at a single timepoint did not predict reported stress four hours later. Additionally, the authors found no effect of gender or sex on these relationships (58). One of the nine studies that examined camouflaging’s relationship with stress failed to find any significant relationship across all analyses (40).

Eight of nine studies showed that higher camouflaging relates to worse stress. While effect sizes differed by analysis, most effect sizes reported were moderate. Overall, these results suggested that camouflaging and stress have a positive relationship with high camouflaging relating to higher stress.

#### Mental wellbeing and quality of life

Six studies examined the relationship between camouflaging and mental wellbeing in an autistic sample and found significant negative results (13, 31, 48, 59–61). When effect sizes were reported they were small to medium and showed greater camouflaging was associated with worse mental wellbeing. One study found mental wellbeing related to camouflaging in a negative quadratic format but also a positive linear format in a Japanese sample (48). Two studies examined the relationship between mental wellbeing and the subscales of camouflaging (13, 29). Assimilation had a weak to moderate negative relationship with mental wellbeing that was stronger than wellbeing’s relationship with camouflaging, masking, or compensation. (13, 29, 52). Additionally, the total camouflaging score had stronger relationships than masking or compensation (13, 29). In one study, compensation had a positive relationship with wellbeing (52, 62). In two of studies that measured mental wellbeing in both autistic and non-autistic samples, the non-autistic sample had descriptively stronger relationships compared to the autistic sample (13, 29). In another, only the autistic group had camouflaging related to psychological quality of life (31). Gender did not moderate the relationship between camouflaging and psychological quality of life (31). In one study’s Japanese sample, mental wellbeing was found to have quadratic and linear relationships with camouflaging. However, Oshima and colleagues suggested this may be due to cultural factors in Japan and may not be generalizable worldwide (48). One of the eight studies to examine mental wellbeing and camouflaging’s relationship failed to find any significant relationship in an autistic sample across all analyses (49, 62).

Overall, seven of eight suggested a negative relationship between mental wellbeing and camouflaging or its subscales. However, given the small sample size of studies this relationship is not as clear as that for anxiety or depression. Given a number of non-significant analysis and a lack of uniformed directionality for compensation’s relationship, the relationship between camouflaging and mental wellbeing is less supported than the relationships between camouflaging and stress, anxiety, and depression.

#### Burnout

Four studies examined burnout’s relationship with camouflaging in an autistic sample and all found significant results (32, 39, 47, 54). While autistic burnout is not yet a diagnosable condition, studies have worked to define and provide measurements of the phenomena (20, 21). While a uniformed definition has not been verified, autistic burnout appears to include feelings of exhaustion and loss of ability in a way that is not equitable with non-autistic work burnout or general depression (20, 21). As such these relationships may be due to overlapping conceptualization or bias during the creation of burnout questionnaires. One study examined personal burnout using a measure not explicitly targeting autistic burnout in addition to examining autistic burnout (47).

When provided, effect sizes were small to medium with greater camouflaging relating to greater burnout. Autistic burnout related to camouflaging in 3 of 4 studies (32, 39, 47). In another study, only one of two autistic burnout measures related to camouflaging (54). Personal burnout related to camouflaging in the one study it was used (47). Subfactors of autistic burnout and personal burnout also had small to moderate associations with camouflaging in two studies, though this was not found for all subfactors in all analyses for said studies (47, 54). Assimilation, masking, and compensation are each related to burnout in an autistic sample and a socially anxious sample, but not a sample of those with dual diagnoses (32). For a socially anxious sample, burnout had a numerically greater relationship with assimilation over compensation and masking (32). For the ASD group, assimilation predicted burnout better than masking or compensation in some, but not all, analyses (32). In one study, the other autistic measure, a pre-publication ABM, was not significantly related with camouflaging (54).

Four studies suggested a connection between camouflaging and burnout, especially between camouflaging and autistic burnout. In one study, for autistic burnout, this relationship existed in both autistic and non-autistic, socially anxious samples.

#### Qualitative results

Sixteen of the forty-eight studies included qualitative analyses relating to camouflaging. This ranged from brief summaries in discussions to direct report of participant perspectives and full thematic or content analysis of the relationship between camouflaging and mental health factors. Mental health issues were mentioned as potential consequences of camouflaging in many studies and as a potential driver of camouflaging in others. In addition to the direct relationship between camouflaging and mental health, other factors such as bullying, isolation, and other mental health factors affected these relationships.

Qualitative studies featured interviews and/or open-ended questions as ways to collect primary data. Nine studies used interviews to collect qualitative data (21, 63–70). One study featured interviews guided by photo-elicitation (67). Seven studies used surveys for collection of qualitative data (8, 20, 25, 33, 40, 65, 71). One study analyzed data using content analysis (40). One study used a Grounded Delphi method (20). Four studies explicitly mentioned using reflexive analysis (63, 66, 68, 70). The remaining qualitative studies used otherwise undefined thematic or qualitative analysis. Ten studies explicitly stated the use of an inductive approach (8, 20, 21, 33, 63, 65–68, 71). Of these, one explicitly mentioned or implied use of deductive methods as well (21). All studies featured data directly from at least one autistic individual, but three also featured data from caregivers and or teachers (64–66). Notably some studies did also analyze previous literature alongside primary data, but only results on primary data were discussed here. Note that we reported findings using the terminology of the reviewed manuscripts. That is, when a reviewed manuscript used the term, “anxiety”, we used that same term in this review, despite the potential conceptual overlap between mental health concepts (e.g. a participant may have reported feeling ‘stress’ despite the description aligning more accurately with ‘anxiety’). Additionally, in this manuscript, low mood is discussed in tandem with depression, worry and fear are discussed with anxiety, and fatigue is discussed with exhaustion and burnout. While these constructs may be distinct, given the conceptual overlap, these results are grouped for integration of results.

#### Depression/low mood

A few papers discussed depression or low mood as having a relationship with camouflaging (8, 25, 53, 63, 67, 68, 71). Multiple studies expressed that camouflaging may directly or indirectly lead to depression, lowered mood or sadness (8, 25, 63, 67, 71). This included participant reports that “‘it can lead to depression’”, that after masking participants felt “‘a bit sad’”, and that “prolonged camouflaging could ultimately lead to issues such as … depression” (63, p. 11; 67, p. 10; 25, p. 37) as well as reports that being able to be authentic led to more positive mood (63, 69). Additionally, participants reported feeling like they are lying to others or being inauthentic via camouflaging and how in turn they felt isolated from others which contributed to feelings of depression (8, 25, 63). Some studies suggested that having to hide one’s authentic self or true interests caused sadness or grief in participants (67). In one study a neurotypical participant reported grieving their loss of self from camouflaging (33). Camouflaging was also suggested to cause lower mood or sadness via consequences of delayed diagnosis (66). In one study lowered mood was suggested to be a possible consequence of failing to camouflage or socialize well due to extended camouflaging and socializing being overwhelming (68). In contrast to camouflaging seeming to contribute to low mood, some studies reported that for some participants, there was a sense of satisfaction or even happiness when camouflaging was done successfully (8, 25, 63, 68). Based on pride some feel regarding their ability to mask, one study suggested that how one “felt about” masking is linked to depression, rather than masking itself (53). In addition to being a potential consequence of camouflaging, depression and low mood acted as possible drivers for camouflaging (63). Notably, these depressive or low mood feelings were, in part, caused by bullying and ostracization (63).

#### Anxiety

A greater number of papers included anxiety, fear, or worry as relating to camouflaging compared to depression in some way (8, 63, 64, 66, 69–71). Anxiety was suggested as an outcome of camouflaging by some papers (8, 63, 66, 69, 71). Aspects contributing to this anxiety included monitoring of self and others, camouflaging of stimming, and predicting their social actions and the actions of others, as well as the general difficulties of camouflaging (8, 63, 66, 69). Anxiety and camouflaging were described as both increasing together in response to social pressure (64). The anxiety caused by camouflaging was reported to get worse when camouflaging affected one’s sense of self (8).

One participant reported it was the time spent just before camouflaging or masking that is most anxiety inducing rather than the act of camouflaging itself, however the authors of that study interpreted this as relating to the environmental context of the camouflaging (63). Further participants similarly reported that anxiety changed with their social or emotional environment and context related to camouflaging (63, p. 9). When able to be authentic and not camouflage, some participants reported feeling more “relaxed and comfortable” and thus less anxious (63). Studies also established a link between anxiety and camouflaging via social uncertainty, with one study mentioning being unconfident of the success of their camouflaging causing “frustration, anger and anxiety” and another reporting how camouflaging was encouraged by anxiety from social uncertainty which camouflaging helped to reduce (8, 66, p. 2081). In this way camouflaging may have caused a decrease in anxiety in some by decreasing uncertainty in their socialization success. In three studies, masking of social anxiety was discussed (63, 66, 70).

Social anxiety and its related factors possibly contributed to camouflaging in other studies, specifically, fear of negative evaluation and worry over bullying (63, 65, 66). Masking as well as internalization of anxiety as an aspect of camouflaging was discussed (63, 64, 66). Masking of emotional factors was reported as contributing to anxiety (63).

#### Stress

In addition to anxiety and depression, stress was related to camouflaging (8, 21, 25, 33, 40, 64, 66–69, 71). Concern over the success of camouflaging attempts and their general social competencies contributed to this relationship beyond direct camouflaging effects increasing stress (8, 66, 68). Stress also affected camouflaging with one participant explaining “‘I can manage to exist in an NT world as long as I’m ok mentally, if I’m stressed … it all turns to jelly, as do I’”, though this was interpreted by the authors as a component of camouflaging’s relationship with exhaustion and burnout (33, p. 334). Two studies described camouflaging leading to identity issues or sense of self issues that caused or worsened stress with one describing it as participants being “stressed in their inner self’ (8, 25, p. 27). Similarly, in another study, disconnection with others due to feeling as though others do not know their true self similarly caused increased stress (33). As with anxiety, extensive monitoring of themselves and others during camouflaging was described as causing increased stress (8). In one study, camouflaging was described as a significant stressor that if left unchecked would result in burnout (21).

General social stress also drove camouflaging and some reported that camouflaging allowed them to minimize their social stress (8). Camouflaging of stress was another commonly reported aspect that deserves further exploration in regards to qualification as “autistic camouflaging” (8, 64). Additionally masking of stress was said to increase stress in one study (64). Finally, choosing not to mask was reported stressful for one participant at first, while others described less defined negative feelings in response to camouflaging, including one comparing it to claustrophobia (69).

#### Burnout/exhaustion

The most commonly reported mental health aspect was exhaustion, also represented as fatigue, burnout, and energy drain (8, 20, 21, 25, 33, 40, 63, 65–71). Extensive attention to oneself, others, and the social situation as well as self-management were specified as aspects of camouflaging contributing to exhaustion (68, 70, 71). In another study, “‘constantly having to be something else’” was a contributor to exhaustion (8, p. 2529).

Clearly associated with exhaustion is the idea of burnout as well as “autistic burnout.” Stressors, particularly camouflaging or “masking” seem to contribute to the start of this burnout (20, 21). Burnout and exhaustion seem to contribute to one another with some studies reporting burnout being due to exhaustion (20, 71) and others expressing exhaustion as an impact of burnout (20). Masking was described in one study defining autistic burnout as the “most prominent life stressor” contributing to burnout, with one participant expressing that one way to mitigate autistic burnout is not camouflaging (21, p. 137). In another study developing a definition for autistic burnout, camouflaging was seen as a key factor contributing to autistic burnout, with a majority of participants (81.8%, n =18) in the initial steps of definition development agreeing that the definition should include “fatigue from camouflaging or masking autistic traits” (20, p. 2363). Although not directly relating camouflaging to mental health, in a study that determined camouflaging as a contributing factor of burn-out, burn-out was associated with anxiety, depression, and difficulties in emotional processing, cognitive processing, exhaustion, self-image, and suicidal ideation. (20). Autistic burnout was associated with poor quality of life, self-harm, self-worth, and suicidality (21). Burnout and exhaustion from camouflaging related to suicidality and other mental health factors with one participant reporting they “‘only get suicidal during meltdowns … I spent 13 years burnt out’” however analysis in neither paper claimed that increase in suicidality was solely through burnout and exhaustion (33, 63, p. 334). Although camouflaging led to burnout, burnout also led to less camouflaging by way of less energy or ability (20). Camouflaging contributing to burnout was shared between autistic and non-autistic subjects (33).

Overall across qualitative studies, direct and indirect positive and negative associations between camouflaging and mental health. While some factors mediating these relationships were discussed, such as bullying, identity, and burn-out, further research is needed to explore the complex nature of these relationships. Importantly, autistic burn-out is associated with exhaustion and has features that overlap with depression (20, 21). As mentioned above, anxiety was presented as a conflicting outcome of camouflaging with camouflaging positively or negatively influencing this factor according to different participants or in different contexts. Despite the above negative effects on mental health, some participants reported positive feelings from camouflaging such as accomplishment, confidence, or relief (8, 63, 68, 71). Additionally, some participants even when acknowledging the negative effects of camouflaging claimed it was worth the risk, depending on the situation (25, 71). In one study the dissonance between desiring to be authentic but still camouflaging was seen to possibly contribute to camouflaging related distress (67). As with depression, distress was an outcome of bullying that drove camouflaging by means of trying to avoid said feeling (63). Additionally, emotional discomfort was mentioned as being associated with camouflaging in one study (70). A common theme in some papers was emotional outbursts or mental breakdowns upon returning to a situation where the person no longer needs to camouflage or simply when the strain or taxing of camouflaging became too much (33, 63, 64, 66, 71). It is unclear in some areas if this was due to exhaustion from autistic camouflaging or simply ceasing to hide emotions when in a safe context. Distress, emotional discomfort, and emotional outbursts associated with camouflaging may also be linked to anxiety, stress, depression, mental wellbeing or burnout. Multiple studies and participants included hiding or faking of emotions or states of mind as a form of camouflaging (8, 63, 65–67, 70). Another included camouflaging of learning challenges as an aspect of camouflaging relating to mental health (66). Notably, while mental health factors were described as potential consequences of camouflaging they were also discussed as possible contributing factors to camouflaging. Chapman et al. (63, p. 5) described this as a “‘self fulfilling prophecy’” when a participant described masking their anxiety as causing further anxiety. Although masking of emotions may not perfectly relate to “autistic camouflaging” the same self-fulfilling prophecy idea still is suggested across these results. Some acknowledged that it was possibly not only the act of camouflaging, but rather camouflaging over an extended period or periods of time that led to the more severe poor mental health outcomes (21, 25, 67, 71). Of note was that specific camouflaging and suppression of stimming were mentioned in two papers contributing to poor mental health such as poor emotional processing and anxiety or coping behaviors (33, 63). Both studies relate this back to the loss of stimming as a tool for self-regulation.

These qualitative results provide further evidence that camouflaging is associated with poor mental health. Anxiety, depression, burnout, stress, and mental wellbeing were all mentioned as being negatively affected by camouflaging, and additionally, mental health issues contributed to camouflaging according to multiple studies. Some participants, such as those in the study by Seers and colleagues, did not “attribute masking behaviours to poor mental health” beyond mentions of exhaustion. Instead, participants linked these mental health struggles to their social difficulties rather than with camouflaging directly (70, p. 36). In one study, non-autistic individuals reported similar consequences to camouflaging as autistic participants with suicidality, addiction, and eating disorders exceptions only reported by autistic participants in one study (33). Camouflaging or masking of mental health and mood was also mentioned by participants which shows a lack of unified definition of camouflaging between researchers and the community.

## Discussion

This review helps elucidate the current state of research into the relationship between camouflaging and depression, anxiety, stress, mental-wellbeing, and burnout. Knowledge about the state of the literature is critical due to the prevalence of mental health conditions and camouflaging in the autistic population (3, 26). Current research shows progress towards understanding the relationship between camouflaging and mental health, but also indicates areas which need further investigation. Quantitative studies as to the relationship between mental health and camouflaging have mixed results, but most suggest that negative mental health and camouflaging relate to one another. Specifically, results across papers suggest that camouflaging relates positively with depression, anxiety (including social anxiety), stress and burnout, and negatively with mental wellbeing with quantitative results generally showing small to moderate sized relationships. In the autistic group, the weakest relationship was between mental wellbeing and camouflaging with effect sizes being almost entirely small or non-significant. The strongest relationships appeared to be between depression and camouflaging and anxiety and camouflaging. While depression anxiety, social anxiety, stress and burnout all had similar ranges of large, moderate, small, and non-significant effect sizes, anxiety and depression had a greater number of analyses with larger effect sizes, though this may in part be due to the commonality of these constructs in the selected studies. Analysis of the camouflaging subscales of the CAT-Q revealed that for depression, anxiety in general, mental wellbeing, burnout, and stress, a stronger relationship was observed with assimilation (attempting to fit in during social settings) than with compensation (mimicking and practiced skills) or masking (actively attempting to suppress ASD traits). Gender or sex appeared to affect this relationship in some (but not all) studies. Non-significant results may be influenced by discrepancy in gender sample sizes in addition to the relationship of interest. Some studies collected gender identity of participants while others collected sex assigned at birth. This may muddle comparison of sample demographics and gender/sex based analyses. Additionally, due to gender and sex being used interchangeably by some persons, it is difficult to be certain whether a study that claimed to collect gender demographics was interested in gender identity or sex assigned at birth. Age, although a covariate or control variable for some studies (15, 29, 42, 45, 46, 48–50, 52, 61), was not quantitatively examined in its effect on camouflaging’s relationship with mental health. Reviewed results suggest that camouflaging is present in a wide range of ages including during developmental years of adolescents (eg. 10-18). Results including analyses over time are currently limited, but initial studies (50, 58) suggest that camouflaging does not predict an increase in mental health difficulties. Further longitudinal studies may be able to clarify the relationship between mental health and camouflaging over time and its directionality.

The same mental health constructs were reported to have similar relationships across qualitative analyses of interviews and surveys. Qualitative studies echoed the quantitative results in suggesting that camouflaging was positively related with depression, anxiety, stress, and burn-out and negatively related with mental wellbeing. In addition to burn-out, more general terms such as exhaustion and fatigue were also used which may be related to, yet partially distinct from, the concept of autistic burn-out. Unique to qualitative analyses were results suggesting the bidirectionality of these relationships. Qualitative results were mixed regarding whether camouflaging was a contributor to or was encouraged by mental health constructs. Ultimately, the results suggest that the relationship was bidirectional or even cyclical, with poor mental health contributing to camouflaging and higher levels of camouflaging in turn contributing to poor mental health. Various psychosocial constructs such as identity and victimization were suggested to be involved in these relationships. These factors may not only mediate the relationship, but also explain aspects of the directionality, (i.e., Being bullied may cause anxiety or low mood; individuals may camouflage to avoid being bullied and corresponding negative mental health outcomes; this in turn may affect sense of self which may lead to inner stress). The qualitative results suggest that camouflaging may be in part driven by mental health factors in addition to external and internal psychosocial factors. Additionally, qualitative studies uniquely suggested that camouflaging and masking, at least in a layman’s understanding, may extend beyond the camouflaging of autistic traits and into masking other conditions and difficulties.

The finding that camouflaging is associated with and may contribute to poor mental health outcomes is significant given that autistic individuals face discrimination and stigma. In the face of such stigma, camouflaging may be a common (or necessary) tool for social and even physical safety. Although some positive outcomes of camouflaging were noted, mental health associated outcomes were largely negative. Thus, the results suggest that caution is warranted when working with autistic individuals if camouflaging may be implicitly or explicitly encouraged. On a societal level, these findings have important implications in the context of the double-empathy problem. This term, coined by Damien Milton, suggests that apparent social-communicative challenges experienced by autistic individuals may be addressed via mutual understanding rather than being conceptualized as deficits in autistic people (Milton, 2012). It could be hypothesized that if neurotypical individuals bore the same social weight as autistic people in cross neurotype communication then autistic individuals may not feel the need to camouflage. Focusing on bridging communication gaps without encouraging camouflaging is necessary to create mutually beneficial environments without sacrificing mental health. As for the clinical importance of these findings, some have questioned the benefit of social skills programs and applied behavior analysis (ABA) therapy, saying that such treatments may encourage masking (41, 72, 73). If such programs increase camouflaging, then mental health may be harmed even as outward behavior (e.g. spoken language, eye contact, gestures) seemingly improves. One study failed to find a difference in camouflaging levels between those who had versus had not previously engaged in ABA (41). However, more research is needed to examine whether interventions for autism relate to camouflaging (73).

Assimilation appeared to have a descriptively stronger relationship with mental health compared to other subscales of camouflaging on the CAT-Q, though no statistical analysis was performed to support or refute this comparison. This relationship may be due to the wording and content of assimilation’s items. Five of the eight assimilation items are related to internal states (e.g. ‘I [don’t] feel’ statements) while compensation and masking are primarily composed of action statements such as “I adjust my body language or facial expressions so that I appear relaxed” (13). Assimilation items thus may better relate to one’s internal state rather than one’s actions and behaviors. Additionally, half of the assimilation items relate to feelings of authenticity or feelings of needing to suppress authenticity (pretending, performing, being oneself, putting on an act). The relevance of inauthenticity and lack of belonging may explain assimilation’s strong relationship with mental health. These factors may better relate to mental health as loneliness and social isolation can be associated with depression and anxiety (74–78). However, findings regarding assimilation are complicated by exploratory factor analysis into the CAT-Q. For example, one study suggested that there was conceptual overlap between the assimilation subscale of the CAT-Q and social autistic factors measured by subscales of the BAPQ (55). Additionally, although not as severe, there was cross-loading between social anxiety items and assimilation items (55). This may suggest some conceptual overlap between these factors. Such overlap may partially contribute to assimilation’s stronger relationship with mental health factors compared to compensation and masking. Importantly, these subscale findings did not include statistical comparisons between subscales’ relationships with mental health factors. As such, caution should be used when interpreting the importance of the assimilation subscale and further research running such statistical analyses may provide more insight into the importance of these sub-constructs.

Alternatively, should the camouflaging scale and its subscales be accurately reflecting these constructs, it would appear that assimilation may be strongly contributing to the relationship between camouflaging and mental health outcomes. This has real world applications in the form of interventions and/or advice offered to autistic individuals seeking to overcome social difficulties. Assimilation should be cautioned by these programs, whereas masking and compensation may be less problematic. In practical terms, a social skills program could center authenticity as it teaches autistic individuals socialization strategies. For example, when teaching socially appropriate ways to start a conversation with a classmate (e.g. using social cues to see if the classmate appears receptive to a conversation, bringing up a topic of mutual interest, etc), the therapist or clinician could emphasize the importance of remaining true to oneself (e.g. not pretending to like things just to make a friend, not feeling pressured to begin a conversation with someone if you dislike them). Of note, however, is that encouragement to camouflage or assimilate may be implicit rather than explicit. Those running such programs may benefit their participants by considering unintentional messaging in addition to providing active encouragement to maintain authenticity. Merely stating that authenticity is important may not be enough to prevent implicit (even if unintended) encouragement of assimilation. Additionally, given the assimilation subscale’s questions regarding avoidance of socialization, forcing socialization, and needing social support, it may be important for those working with autistic individuals to focus not only on knowledge of social skills but also practicing them in a way where the client feels authentic and confident. By reminding autistic individuals about the importance of being their authentic selves–even in the context of an intervention–such programs could ensure participants have tools and strategies to socialize with peers while feeling confident to ‘be themselves’.

There are multiple possibilities for why camouflaging seemed to have a similar relationship with each mental health factor (e.g. depression, anxiety) rather than unique relationships with each diagnosis or mental health condition. First, mental health conditions often co-occur, with one study finding nearly one third of Americans have co-occurring mental disorders, and mental health measures in some studies correlating with one another. A second possibility is that a single factor such as autistic burnout or autistic traits is the overarching concept connecting these individual factors. However, given that the relationship between mental health and camouflaging is present in the non-autistic population, even when measuring camouflaging using the same measures in both groups, it is more likely that any such factor would be something applicable to the general population, such as authenticity or acceptance. While descriptive results suggest the relationship may be even stronger in non-autistic samples, given the lack of between group analysis and differing sample sizes by diagnosis, caution is warranted. Qualitative results bolster this hypothesis, as they included reports of “masking” emotions and mental health, rather than autistic traits alone. Taken together, these findings suggest that social camouflaging may not be unique to autistic individuals, but rather may be composed of impression management, emotional regulation, or other constructs which occur in the general population. This is further supported by how in the few studies who examined them alongside an autistic sample, even those who were without any developmental or mental health condition still appear to have the association between camouflaging and mental health (28, 33). Alternatively, if camouflaging is unique to autism, it would suggest that current camouflaging measures assess something other than “true” camouflaging. Future research should continue to explore measures of camouflaging and mental health in non-autistic and autistic samples, along with samples of individuals with other diagnosed mental health conditions (e.g. major depressive disorder, social anxiety, etc), while also considering how camouflaging and its related sub concepts are defined and measured. This will help to not only better establish the separation of camouflaging from other constructs but also clarify what aspects of camouflaging are unique to the autistic population.

Various factors may be responsible for discrepancies in results of studies analyzing the same constructs. For one, geography of studies and nationality of participants varied from study to study, although most studies were North American, European, or Australian. Cultural differences may affect levels of camouflaging, cultural perception of camouflaging, and social consequences of camouflaging all of which may interact with how camouflaging and mental health interrelate. Furthermore, other demographic differences such as sample gender and sex ratios, diagnostic comorbidities, education levels, and other factors may further contribute to differences in study results. While most quantitative studies used the same measure for camouflaging, individual measures for mental health varied more frequently. As such, while studies may target the same constructs (e.g. camouflaging, anxiety, etc), they may arrive at different conclusions based on the measure used. This is especially true when measures may be designed via different diagnostic standards (e.g. based on guidelines from differing manuals, years, or focused on differing aspects of the constructs) or are for more novel constructs like autistic burnout.

This review is not without its limitations. Not all articles were able to be extracted by multiple researchers. Quantitative mental health outcomes outside of depression, anxiety, stress, mental wellbeing, and burnout were not reported, nor were results from broad measures of combined mental health (e.g. measures which target ‘mental health’ as a broad construct). This review is limited in that only direct relationships were reported (e.g. analyses including mediation models were not reported). Future reviews and studies may benefit from investigating possible mediating factors and examining effects of age to explore developmental effects of the relationship between camouflaging and mental health. Despite these limitations, this review establishes an overview of the relationship between camouflaging and mental health.

## Conclusion

Overall, results suggest that there is a negative relationship between camouflaging and mental health. Specifically, higher levels of camouflaging were associated with stronger depression, anxiety, stress, and burnout symptoms and worse mental wellbeing. Interestingly, both non-autistic and autistic individuals exhibit similar relationships between mental health and camouflaging. Qualitative research suggests that these relationships may be bidirectional, while quantitative results show that individual subscales of camouflaging may have uneven influence over mental health outcomes. Future studies may benefit from examination of directionality in the relationship between camouflaging and mental health along with potential mediating factors. Longitudinal methods may help clarify directionality and how this relationship might change across development in both autistic and non-autistic populations.

## Data Availability

The original contributions presented in the study are included in the article/**Supplementary Material**. Further inquiries can be directed to the corresponding author.
