# Supplementary material for: The relationship between autistic camouflaging and mental health: a scoping review
Source: Front Psychiatry. 2026 Jun 2;17:1701615. doi: 10.3389/fpsyt.2026.1701615 (PMC13270291; doi:10.3389/fpsyt.2026.1701615)
Supplement: Supplementary file 1 [file DataSheet1.pdf]

CODING TEMPLATE

Source:

Study ID:	
Review Author ID:	Your initials
Citation/Contact Details:	Just citation APA
Publish Year:	

Eligibility:

Confirm Eligibility:	
Reason for Exclusion:	If any

Methods:

Study Design:	Objectives/General aims:<br><br>Methods (general and analytical):<br><br>Hypotheses/Research Questions/Specific Aims:
Total Study Duration:	If no duration is given see if a time window is given for recruitment. Specify if study duration or recruitment period duration
Sequence Generation:	Were tasks/measures/etc counterbalanced, randomized, etc
Allocation Sequence Concealment:	Blinding subject to sequence generation (IF CLINICAL TRIAL or put not applicable)
Blinding:	If applicable
Other concerns about bias: (or things done right)	Power and limited effect sizes, Community based participatory research, limited sample size, other mentioned limitations etc

Participants:

Total Number (initial):	Total number of participants<br>List and label both initial and final
Setting:	Where were participants recruited from?<br>What sort of recruitment? convenience sampling?<br>Other important setting factors, ie. all participants were enrolled in a medical home model practice.
Diagnostic Criteria:	What was the criteria for diagnosis (official diagnosis or

	self-id? DSM-5? Confirmed by ADOS? Does their autism diagnosis include Ausbergers and PDD-otherwise not specified?)
Diagnosis:	What diagnosis were necessary to participate or separates groups<br><br>What other diagnosis were collected
Age(s):	Mean, Standard deviation, and range of ages if available<br><br>What were the age inclusion/exclusion criteria if listed
Sex:	number/% of each sex or gender<br><br>Note if gender identity or sex assigned were used
Country:	Breakdown of where participants live/nationality<br><br>Also list where study took place

CODING TEMPLATE

Interventions:

Total # of Groups:	If intervention or multiple groups
Specific Intervention:	If intervention
Intervention Details:	If intervention

Outcomes:

Outcome Definition:	What was measured/assessed?
Unit of Measurement:	How was it measured/assessed (what scale or what survey, etc)

Results:

# of participants per group? (if eligible)	If intervention or if multiple groups in general
Final Sample Size	FINAL sample size
Missing participants (# and reason)	
Summary of data (means, SD's, table)	Means and SD of any data mentioned in text. All tables from text.
Estimate of effect (f value, include p value, etc)	F value, R value, p value, any other analysis results. Include even if also in a table in previous box
Subgroup analysis (if applicable)	
