# Supplementary material for: The relationship between autistic camouflaging and mental health: a scoping review
Source: Front Psychiatry. 2026 Jun 2;17:1701615. doi: 10.3389/fpsyt.2026.1701615 (PMC13270291; doi:10.3389/fpsyt.2026.1701615)
Supplement: Supplementary file 2 [file Table1.docx]

**Table S1**

*General Study Information* (Studies were not uniform in use of gender identity or sex assigned at birth. The other gender column includes nonbinary, “prefer not to answer” or a similar option, genderfluid, demifemale, grouped transgender identities, and “other” or similar options. NR = details regarding this data were not reported. ^a^ Gender was reported if available. ^b^ Combined diagnosed autistic and suspected autistic groups. ^c^ Only percent male reported. ^d^ Some additional participants were guardians or teachers of autistic youth. Gender and age reported here are of the autistic participant(s). ^e^ Includes those with dual autism and social anxiety diagnoses. The reported total sample did not equal separate groups added together. For gender calculations, a reported total of 254 participants was used. ^f^ Provided gender identified 21 responses despite there being only 19 participants. Reported sample of 19 was used for gender percentage calculation and as such percentages add up to more than 100%. ^g^ It was unclear whether this was based on the initial sample or final sample for the source paper. ^h^ Means and standard deviation estimates calculated based on values provided for individual subsamples (ie. based on gender, country, and/or diagnosis) using <https://www.statstodo.com/CombineMeansSDs.php>. ^i^Study included two time points and thus two sets of age data. ^j^ Data of total subsample used for relevant analyses are reported here.)

|  | Non-ASD Participants (n) | ASD Participants (n) | % ASD Formally Diagnosed | Total Sample Age Range | Total Sample Age Mean (SD) | Total Sample Gender/Sex: %F, %M, %Other^a^ | Gender or sex or both or NR or unclear | Nationality/ Geography |
| --- | --- | --- | --- | --- | --- | --- | --- | --- |
| Arnold et al., 2023 | 0 | 141 | 100% | 20.3–71.0 | 40.2 (10.8) | 64, 16, 20 | gender | AUS+ |
| Atkinson et al., 2025 | 0 | 72 | 83.33% | 11–16 | 13.37 (1.42) | 34.72, 54.17, 11.11 | both | UK & IRE |
| Attaulah et al., 2023 | 0 | 400 | 100% | 18–44 | NR | 61, 29.5, 9.5 | gender | PAK |
| Benatov et al., 2025 | 0 | 92 | NR | 19–70 | 33.43 (10.7)^g^ | 60.87, 11.96, 27.17 | gender | NR |
| Bradley et al., 2021 | 0 | 277 | 74.37% | adults | 66.74 (48.35)^h^ | 66.43, 33.57 | gender | UK+ |
| Cage & Troxell-Whitman, 2019 | 0 | 262 | 100% | 18–66 | 33.62 (11.52) | 51.53, 42.37, 6.12 | gender | NR |
| Cage et al., 2018 | 0 | 111 | 90.09% | 18–72 | 36.4 (12.0) | 60, 27, 14 | gender | UK+ |
| Cage et al., 2022 | 0 | 196 | 65.82% | 18–67 | 32.47, (11.15) | 63.27, 14.80, 21.94 | gender | UK, USA, AUS/NZ, CAN, other EUR,Asia |
| Cassidy et al., 2018 | 169 | 164 | 100% | 20–60 | 40.33 (10.94)^h^ | 64.26, 35.74 | sex | UK+ |
| Cassidy et al., 2021 | 268 | 421^b^ | 73.16% | 18+ | 40.53 (13.91)^h^ | X,  28.88, X^c^ | both | UK, Other |
| Chapman et al., 2022 | 0 | 20 | 100% | 13–19 | 15 (NR) | 50, 50, 0 | gender | ENG |
| Conde-Pumpido Zubizarreta et al., 2025^j^ | 337 | 103 | 100% | 18-64 | 33.26 (10.88) | 74.55, 19.55, 5.91 | gender | SPN |
| Corscadden et al., 2021 | –^d^ | 1 | NR | 19 | 19 (0) | 100, 0, 0 | NR | NR |
| Drapalik et al., 2025 | 0 | 10 | NR | 11–16 | 13.1 (1.52) | 70, 0, 30 | gender | Likely USA |
| Evans et al., 2024 | 0 | 342 | 57% | 18–80 | 38.46 (11.41) | 60.72, 10.81, 28.38^e^ | gender | NR |
| Evans, 2022 | 0 | 248 | 54.84% | 18–70+ | NR | 70.44, 10.12, 19.43 | gender | NA, EUR, OCE, SA, AFR, Asia |
| Galvin et al., 2024 | 0 | 294 | 100% | 18–65 | 30.53 (12.57) | 51.70 48.30 | sex | UK |
| Gonçalves, et al., 2025 | 0 | 253 | 63.60% | 18–72 | 33.1 (10.1) | 67.59, 24.90, 6.72 | gender | Brazilian |
| Halsall et al., 2021 | –^d^ | 8 | 100% | 12.08–15.16 | 13.58 (0.93) | 100, 0, 0 | gender | UK |
| Higgins et al., 2021 | 0 | 23 | 100% | 27–64 | 42.8 (9.9) | 69.57, 26.09, 4.34 | gender | Australia |
| Hollocks et al., 2025 | 0 | 806 | 99.63% | 18.2-83.3 | 40.2 (13.8) | 51.6, 39.2, 9.2 | both | US |
| Hongo et al., 2024 | 410 | 204 | 100% | 20–65 | 42.15 (12.91) | 48.05, 51.95, 0 | sex | Japan |
| Howe et al., 2023 | 0 | 8 | 100% | 10–14 | 11.88 (1.89) | 37.50, 62.50, 0 | gender | CAN |
| Hull et al., 2017 | 0 | 92 | 100% | 18–79 | 43.09 (15.09)^h^ | 59.78, 32.61, 7.61 | gender | BRI, NA, W. EUR, Other |
| Hull et al., 2019 | 478 | 354 | 100% | 16–82 | 36.01 (14.84) | 52.16, 36.06, 11.78 | gender | UK + |
| Hull et al., 2021 | 0 | 305 | 100% | 18–75 | 41.90 (NR) | 59.34, 34.10, 5.90 | gender | BRI; NA, EUR, AUS, Asia, ME, SA |
| Keating et al., 2024 | 0 | 306 | 100% | adult | 30.72 (9.15)^h^ | 43.46, 42.81, 13.73 | both | AUS, BEL, AUS, JAP, |
| Khudiakova et al., 2024 | 0 | 409 | 34.96% | 18–67 | 31.78 (11.00) | 45.48, 42.05, 12.47 | gender | UK, POL, S. AFR, ITA, US, CAN, Other |
| Lai et al., 2017 | 0 | 60 | 100% | 18–49 | 27.2 (7.3) | 50.00, 50.00, 0 | sex/gender | UK |
| Liu et al., 2023 | 105^d^ | 100 | 100% | 12–18 | 14.70 (1.76)^h^ | 32.68, 67.32 | sex | Taiwan |
| Mantzalas et al., 2024 | 0 | 238 | NR | 18–75 | 37.89 (11.38) | 71.43, 17.23, 11.34 | gender | AUS, UK, USA, NZ, Other |
| Mckinnon et al., 2024 | 0 | 308 | 86.04% | 18–76 | 36.17 (11.62) | 61.36, 28.57, 10.06 | gender | NR |
| Miller et al., 2021 | 94 | 144 | N/A | 18-74 | 36.00 (11.52)^h^ | 76.05, 14.71, 9.24 | gender | NR |
| Milner et al., 2023 | 357 | 78N | 22.22% | 18.43–28.12 | 22.40 (NR)^h^ | 60.23, 39.77, 0 | sex | UK + |
| Moore et al., 2023 | 0 | 627 | 59.65% | 18–70 | 34.15 (12.96) | 52.00, 19.30, 28.71 | gender | NR |
| Oshima et al., 2024 | 0 | 515 | 100% | 18-75 | 40.15 (NR)^h^ | 52.24, 44.25, 3.51^g^ | mixed | UK, JAP, + |
| Perry et al., 2022 | 0 | 223 | 100% | 18–65 | 34.19 (11.00) | 58.30, 23.77, 17.94 | gender | UK, NA, other EUR, AUS, NZ, UA, + |
| Pyszkowska, 2024 | 65 | 193^a^ | 100% | adult | 28.45 (7.03)^h^ | 73.23, 11.02, 16.93 | gender | POL |
| Pyszkowska et al., 2025 | 0 | 152 | 99.98% | 18-52 | 28.55 (6.60) | 73.68, 12.50, 13.82 | gender | POL |
| Raymaker et al., 2020 | 0 | 19 | 100% | 21–65 | 37.32 (22.85)^h^ | 52.53, 42.11, 21.05^f^ | gender | US |
| Rehman et al., 2023 | 0 | 90 | NR | 18–30+ | NR | 45.56, 54.44, 0 | gender | PAK |
| Sandland, 2018 | 0 | 6 | 100% | 11–18 | NR | 100, 0, 0 | NR | NR |
| Scheeren et al., 2025 | 0 | 87 | 100% | 17.90–68.90 | 48.46 (12.78) | 54.0, 33.3, 12.6 | both | NETH |
| Schuck et al., 2019 | 0 | 28 | 100% | 18-55 | 26.9.2 ((8.34)^h^ | 39.29, 60.71, 0 | gender | NR |
| Seers & Hogg, 2022 | 0 | 8 | 100% | 24–54 | 39.12 (9.3) | 100, 0, 0 | gender | AUS |
| Tamura et al., 2025 | 0 | 287 | 100% | 20–63 | 37.52 (9.81) | 41.81, 50.87, 7.32 | gender | JAP |
| van der Putten et al., 2025 | 0 | 322 | 100% | 30-84; 32-86^i^ | 52.4 (12.4); 54.3 (12.3)^i^ | 47.29, 52.41, 0.30 | sex | NETH+ |
| White et al., 2024 | 0 | 412 | 52.18% | 18–66 | R | 14.32, 30.34, 79.61 | gender | NR |

**Table S2**

*Measures Collected For Reported Quantitative Results (*PHQ-9 = Patient Health Questionnaire-9; BDI-II = Beck's Depression Inventory-II; DASS-21 =Depression, Anxiety and Stress Scale - 21; BDI = Beck's Depression Inventory; HADS = Hospital Anxiety and Depression Scale; RCADS = Revised Child Anxiety and Depression Scale; BAI = Beck Anxiety Inventory; ASC-ASD = Anxiety Scale for Children- ASD; GAD-7 = General Anxiety Disorder-7; ASA-A = The Anxiety Scale for Autism – Adults; SIAS-6 =Social Interaction Anxiety Scale-6; SPS-6 = Social Phobia Scale-6; LSAS = Liebowitz Social Anxiety Scale; SPIN = Social Phobia Inventory; PSS = Perceived Stress Scale; SAM = Stress Autism Mate, WEMWBS = Warwick-Edinburgh Mental Wellbeing Scales; WHO-QoL Bref-Psychological =World Health Organization WHOQOL-BREF quality of life assessment psychological subscale; PBA-E = Parental Burnout Assessment exhaustion subscale; ABSI = Autistic Burnout Severity Items, ABM = AASPIRE Autistic Burnout Measure; CBI = Copenhagen Burnout Inventory. ‘-J’ indicates a Japanese translation or variant. ‘-Ch’ indicates a Chinese translation or variant. ‘-P’ Indicates a Polish translation or variant. ‘-ES’ indicates a Spanish translation and cultural variant for Spain. ‘-NL’ indicates a Dutch translation or variant.)

|  | Depression | Anxiety | Social Anxiety | Stress | Mental Wellbeing | Burnout | Camouflaging |
| --- | --- | --- | --- | --- | --- | --- | --- |
| Arnold et al., 2023 | PHQ-9 | — | — | — | — | ABSI, pre-publication ABM, | CAT-Q |
| Atkinson et al., 2025 | — | ASC-ASD | — | — | — | — | adapted CAT-Q |
| Attaulah et al., 2023 | — | — | — | — | WEMWBS | — | CAT-Q |
| Benatov et al., 2025 | PHQ-9 | — | — | — | — | Adapted PBA-E | CAT-Q |
| Cage & Troxell-Whitman, 2019 | DASS-21 | DASS-21 | — | DASS-21 | — | — | CAT-Q, custom questionnaire |
| Cage et al., 2018 | DASS-21 | DASS-21 | — | DASS-21 | — | — | Report vs Lack of Report of Camouflaging |
| Cage et al., 2022 | — | — | — | — | WEMWBS | — | CAT-Q |
| Cassidy et al., 2018 | Not Stated | — | — | — | — | — | Custom Questionnaire |
| Cassidy et al., 2021 | PHQ-9 | ASA-A | — | — | — | — | CAT-Q |
| Conde-Pumpido Zubizarreta et al., 2025 | PHQ-9 | GAD-7 | — | — | — | — | CAT-Q-ES |
| Evans et al., 2024 | BDI-II | GAD-7 | — | — | — | — | CAT-Q |
| Evans, 2022 | BDI-II | BAI | — | — | — | — | SVS |
| Galvin et al., 2024 | PHQ-9 | GAD-7 | LSAS | — | — | — | CAT-Q |
| Gonçalves, et al., 2025 1 | DASS-21 | DASS-21 | — | DASS-21 | — | — | CAT-Q |
| Hollocks et al., 2025 | PHQ-9 | GAD-7 | — | PSS | — | — | CAT-Q |
| Hongo et al., 2024 | J-PH-9 | J-GAD-7 | LSAS-J | — | WEMWBS-J | — | CAT-Q-J |
| Hull et al., 2019 | PHQ-9 | GAD-7 | LSAS | — | WEMWBS | — | CAT-Q |
| Hull et al., 2021 | PHQ-9 | GAD-7 | LSAS | — | — | — | CAT-Q |
| Keating et al., 2024 | DASS-21 | DASS-21 | — | DASS-21 | — | — | CAT-Q |
| Khudiakova et al., 2024 | CES-D | — | — | — | — | — | CAT-Q |
| Lai et al., 2017 | BDI | BAI | — | — | — | — | Discrepancy (ADOS vs RMET+AQ) |
| Liu et al., 2023 | — | — | — | PSS-Ch | — | — | CAT-Q-Ch (caregiver) and CAT-Q-Ch (self-report) |
| Mantzalas et al., 2024 | PHQ-9 | GAD-7 | — | DASS-21 | — | ABM, CBI | CAT-Q |
| Mckinnon et al., 2024 | — | — | SIAS-6 and SPS-6 | — | — | — | CAT-Q |
| Milner et al., 2023 | — | — | — | — | WHO-QoL Bref-Psychological | — | prototype CAT-Q |
| Moore et al., 2023 | HADS | HADS | — | — | WEMWBS | — | CAT-Q |
| Oshima et al., 2024 | J-PHQ-9 + PHQ-9 | GAD-7-J and GAD-7 | LSAS-J and LSAS | — | WEMWBS-J | — | CAT-Q-J and CAT-Q |
| Perry et al., 2022 | — | — | — | — | WEMWBS | — | CAT-Q |
| Pyszkowska, 2024 | — | — | LSAS | — | — | ABM-P | CAT-Q |
| Scheeren et al., 2025 | — | — | — | SAM | — | — | Custom Question |
| Schuck et al., 2019 | — | — | SPAI (social phobia) | — | — | — | Discrepancy (ADOS vs AQ) |
| Tamura et al., 2023 | PHQ-9-J | GAD-7-J | LSAS-J | — | WEMWBS-J | — | CAT-Q-J (25 item version) |
| van der Putten et al., 2025 | SCL-90-R | SCL-90-R | — | — | — | — | CAT-Q-NL |
| White et al., 2024 | DASS-21 | DASS-21 | — | DASS-21 | — | — | CAT-Q |

**Table S3**

*Table of ASD Sample Correlations with Camouflaging* (NR = sample size was not reported separately from study sample total; N = no adjustment or covariates noted; B = Bonferroni corrections performed; C = covariates or controlled variables present;* <.05, ** <.01, *** <.001; ^a^ Sex analysis revealed camouflaging to only relate to depression in the male sample (*r* = .53**); ^b^ Bivariate correlation/ partial correlation when controlling for autistic social traits; ^c^ When controlling for Age and Sex; ^d^ Coefficient of Determination reported; ^e^ Pearson’s correlation coefficient reported; ^f^ Spearman’s rho reported; ^g^ Correlation coefficient type not specified in source text); ^h^ Sample size after multiple imputations.

|  | Depression | Anxiety | Social Anxiety | Stress | Mental Wellbeing | Burnout | Sample Size | Adjusted/Controlled |
| --- | --- | --- | --- | --- | --- | --- | --- | --- |
| Arnold et al., 2023^g^ | n.s. | — | — | — | — | .34**;n.s. | NR | N |
| Atkinson et al., 2025^g^ | — | .55** | — | — | — | — | NR | N |
| Attaulah et al., 2023^g^ | — | — | — | — | -.26* | — | NR | N |
| Benatov et al., 2025^e^ | .45*** | — | — | — | — | .26* | NR | N |
| Cage et al., 2022^e^ | — | — | — | — | -.19** | — | NR | N |
| Cassidy et al., 2018^f^ | n.s. | n.s. | — | — | — | — | NR | N |
| Cassidy et al., 2021^f^ | .21** | .45** | — | — | — | — | NR | N |
| Conde-Pumpido Zubizarreta et al., 2025^f^ | .37*** | .36*** | — | — | — | — | 103 | N |
| Evans et al., 2024^g^ | .31* | .37* | — | — | — | — | NR | N |
| Galvin et al., 2024^e^ | .50***^c^ | .52***^c^ | .61***^c^ | — | — | — | NR | C |
| Hollocks et al., 2025^g^ | .29* | .31* | — | 0.25* | — | — | NR | N |
| Hongo et al., 2024^e^ | .18* | .19** | .27** | — | n.s. | — | 204^h^ | N |
| Hull et al., 2019^g^ | .28*** | .35*** | .44*** | — | -0.16* | — | 306 | N |
| Hull et al., 2021^g^ | .22*** | .32*** | .35*** | — | — | — | 305^h^ | N |
| Khudiakova et al., 2024^g^ | .35*** | — | — | — | — | — | NR | N |
| Lai et al., 2017^e^ | .30*^a^ | n.s. | — | — | — | — | NR | N |
| Liu et al., 2023^d^ | — | — | — | .14*** | — | — | NR | N |
| Liu et al., 2023 (parent report CAT-Q)^d^ | — | — | — | .11*** | — | — | NR | N |
| Mantzalas et al., 2024^f^ | .24** | .27** | — | .26** | — | .36**; .30 | NR | N |
| Mckinnon et al., 2024^e^ | — | — | .45***; .33***^b^ | — | — | — | NR | N; C |
| Oshima et al., 2024^e^ | .18** | .14* | .19** | — | n.s. | — | 210 | N |
| Perry et al., 2022^g^ | — | — | — | — | -0.16* | — | NR | N |
| Schuck et al., 2019 | — | — | n.s. | — | — | — | NR | B |
| Tamura et al., 2023^g^ | .28*** | .35*** | .29*** | — | n.s. | — | NR | N |
| White et al., 2024^f^ | .26*** | .35*** | — | .40*** | — | — | NR | B |

**Table S4**

*ASD Sample Camouflaging Subscale Correlations* (NR = sample size was not reported separately from study sample total; N = no adjustment or covariates noted; B = Bonferroni corrections performed; C = covariates or controlled variables present;* <.05, ** <.01, *** <.001; ^a^ Pearson’s correlation coefficient reported. ^b^ Correlation coefficient type not specified in source text. ^c^ Pearson’s coefficient of determination reported. ^d^ Spearman’s rho reported). ^e^ Sample size after multiple imputations.)

|  | Assimilation | Compensation | Masking | Compensation-Masking | Sample Size | Adjusted/Controlled |  |
| --- | --- | --- | --- | --- | --- | --- | --- |
| Conde-Pumpido Zubizarreta et al., 2025^d^ | .47*** | .27* | n.s. | — | 103 | N | Depression |
|  | .44*** | .27* | n.s. | — | 103 | N | Anxiety |
| Hongo et al., 2024^a^ | 0.32** | n.s. | n.s. | — | 204^e^ | N | Depression |
|  | 0.27** | n.s. | n.s. | — | 204^e^ | N | Anxiety |
|  | 0.47** | n.s. | n.s. | — | 204^e^ | N | Social Anxiety |
|  | -0.29** | — | — | — | 204^e^ | N | Mental Wellbeing |
| Hull et al., 2019^c^ | 0.35*** | 0.18** | 0.16** | — | 306 | N | Depression |
|  | 0.41*** | 0.25*** | 0.20*** | — | 306 | N | Anxiety |
|  | 0.60*** | 0.30*** | 0.19** | — | 306 | N | Social Anxiety |
|  | − 0.37*** | n.s. | n.s. | — | 306 | N | Mental Wellbeing |
| McKinnon et al., 2024^c^ | 0.54[***](https://journals.sagepub.com/doi/10.1177/13623613241287964#table-fn8-13623613241287964) | 0.34[***](https://journals.sagepub.com/doi/10.1177/13623613241287964#table-fn8-13623613241287964) | 0.25*** | — | NR | N | Social Anxiety |
| McKinnon et al., 2024 ^c^ (Control for autistic social traits) | 0.25[***](https://journals.sagepub.com/doi/10.1177/13623613241287964#table-fn8-13623613241287964) | 0.25[***](https://journals.sagepub.com/doi/10.1177/13623613241287964#table-fn8-13623613241287964) | 0.30[***](https://journals.sagepub.com/doi/10.1177/13623613241287964#table-fn8-13623613241287964) | — | NR | C | Social Anxiety |
| Moore et al., 2023^a^ | 0.321[**](https://onlinelibrary.wiley.com/doi/10.1002/aur.3073#aur3073-note-0003_118) | n.s. | n.s. | — | NR | N | Depression |
|  | 0.359[**](https://onlinelibrary.wiley.com/doi/10.1002/aur.3073#aur3073-note-0003_117) | 0.231[**](https://onlinelibrary.wiley.com/doi/10.1002/aur.3073#aur3073-note-0003_112), | 0.173** | — | NR | N | Anxiety |
|  | −0.391[**](https://onlinelibrary.wiley.com/doi/10.1002/aur.3073#aur3073-note-0003_120) | n.s. | n.s. | — | NR | N | Mental Wellbeing |
| Liu et al., 2023^c^ | 0.276*** | — | — | 0.041* | NR | N | Stress |
| Liu et al., 2023 (parent report CAT-Q)^c^ | 0.122*** | — | — | 0.051*** | NR | N | Stress |

**Table S5**

*Non-ASD Sample Correlations with Camouflaging* (NR = sample size was not reported separately from study sample total; N = no adjustment or covariates noted; B = Bonferroni corrections performed; C = covariates or controlled variables present.; * <.05, ** <.01, *** <.001; ^a^ Pearson’s correlation coefficient reported. ^b^ Spearman’s rho reported. ^c^ Correlation coefficient type not specified in source text. ^d^ Pearson’s coefficient of determination reported.)

|  | Depression | Anxiety | Social Anxiety | Stress | Mental Wellbeing | Burnout | Sample Size | Adjusted/Controlled |
| --- | --- | --- | --- | --- | --- | --- | --- | --- |
| Cassidy et al., 2021^b^ | .40** | .57** | — | — | — | — | NR | N |
| Conde-Pumpido Zubizarreta et al., 2025^b^ | .54*** | .38*** | — | — | — | — | 337 | N |
| Hongo et al., 2024a | 0.35** | 0.34** | 0.28** | — | -0.22** | — | 410; 318 | N |
| Hull et al., 2019^c^ | — | — | 0.60*** | — | -.43*** | — | 400 | N |
| Liu et al., 2023^d^ | — | — | — | .276*** | — | — | NR | N |
| Liu et al., 2023^d^ (parent report CAT-Q) | — | — | — | .090** | — | — | NR | N |

**Table S6**

*Non-ASD Camouflaging Subscale Correlations* ( NR = sample size was not reported separately from study sample total; N = no adjustment or covariates noted; B = Bonferroni corrections performed; C = covariates or controlled variables present.; * <.05, ** <.01, *** <.001;; ^a^ Pearson’s correlation coefficient reported. ^b^ Correlation coefficient type not specified in source text. ^c^ Pearson’s coefficient of determination reported. ^d^ Spearman’s rho reported.)

|  | Assimilation | Compensation | Masking | Compensation-Masking | Sample Size | Adjusted/Controlled |  |
| --- | --- | --- | --- | --- | --- | --- | --- |
| Conde-Pumpido Zubizarreta et al., 2025^d^ | .53*** | .44*** | .45*** | — | 337 | N | Depression |
|  | .38*** | .30*** | .33*** | — | 337 | N | Anxiety |
| Hongo et al., 2024^a^ | 0.39** | 0.27** | 0.24** | — | 410 | N | Depression |
|  | 0.37** | 0.27** | 0.23** | — | 410 | N | Anxiety |
|  | 0.42** | 0.15** | 0.13** | — | 410 | N | Social Anxiety |
|  | -0.37** | -0.13* | n.s. | — | 318 | N | Mental Wellbeing |
| Hull et al., 2019^b^ |  |  |  | — | 400 | N | Anxiety |
|  | 0.69*** | 0.46*** | 0.35*** | — | 400 | N | Social Anxiety |
|  | — | — | — | — | 400 | N | Stress |
|  | − 0.53*** | − 0.31*** | − 0.24*** | — | 400 | N | Mental Wellbeing |
| Liu et al., 2023^c^ | .339*** | — | — | .161*** | NR | N | Stress |
| Liu et al., 2023 (parent report CAT-Q)^d^ | .117*** | — | — | .050* | NR | N | Stress |

**Table S7**

Regression Analyses in ASD sample (* <.05, ** <.01, *** <.001; NR  = Not Reported; N = no adjustment or covariates noted; B = Bonferroni corrections performed; C = covariates or controlled variables present; M = direct effect in a mediation analysis. ^a^ Unstandardized coefficients were reported in the original publication. Standardized coefficient was calculated for presentation here. ^b^ Standard deviations used for calculating standardized coefficient were combined from male and female subsamples using <https://www.statstodo.com/CombineMeansSDs.php> to calculate standard deviation estimates. ^c^ Effect Sizes of Linear Mixed Modeling were unavailable. ^d^ Additional steps adding quadratic relationships with CAT-Q score and gender interactions are not included as they did not greatly improve explanation of variance (delta R-squared ≤ .01). ^e^ Sample size after multiple imputations.)

|  | Depression | Anxiety | Stress | Social Anxiety | Mental Wellbeing | Burnout | Sample Size | Adjusted/Controlled for Covariates | Regression Type |
| --- | --- | --- | --- | --- | --- | --- | --- | --- | --- |
| Arnold et al., 2023 | — | — | — | — | — | n.s.; 0.00-.22** | 76; 108; 106; 142 | C | Linear |
| Atkinson et al., 2025^a^ | — | .41*** | — | — | — | — | YNR | M | Mediation Analysis |
| Attaulah et al., 2023 | — | — | — | — | -.15*** | — | NR | M | Mediation Analysis |
| Benatov et al., 2025 | NR | — | — | — | — | NR | NR | M | Mediation Analysis |
| Cage et al., 2022^a^ | — | — | — | — | -.22** | — | NR | M | Mediation Analysis |
| Evans et al., 2024 | .31*** | .37*** | — | — | — | — | NR | Boferroni | Linear |
| Galvin et al., 2024^a^ | .85*** | .94*** | — | .18*** | — | — | NR | Age+sex; M | Mediation Analysis |
| Hollocks et al., 2025 | .24*** | .25*** | — | — | — | — | 786; 789 | M | Mediation Analysis |
| Hull et al., 2021^d^ | n.s.; 0.12* | .22*** | — | .19-.21*** | — | — | 286; 305^e^ | C | Hierarchical regression |
| Khudiakova et al., 2024^a^ | .37-.40*** | — | — | — | — | — | NR | M | Mediation Analysis |
| Keating et al., 2024 | NR | NR | NR | — | — | — | NR | C | Hierarchical regression |
| Milner et al., 2023^a^ | — | — | — | — | -.03*^b^ | — | 73 | C; B | Hierarchical regression |
| Oshima et al., 2024 (Japan; Linear) | n.s. | n.s. | — | n.s. | -.15*-.19** | — | 210 | C | Hierarchical regression |
| Oshima et al., 2024 (Japan; Quadratic) | 0.12* | .17** | — | n.s. | -.13* | — | 210 | C | Hierarchical regression |
| Oshima et al., 2024 (UK; Linear) | n.s. | n.s. | — | n.s. | — | — | 305 | C | Hierarchical regression |
| Oshima et al., 2024 (UK; Quadratic) | n.s. | n.s. | — | n.s. | — | — | 305 | C | Hierarchical regression |
| Perry et al., 2022 | — | — | — | — | n.s. | — | NR | C | Multiple Regression |
| Scheeren et al., 2025^c^ | — | — | .56*** | — | — | — | NR | C | Hierarchical regression; negative binomial generalized linear mixed model |
| Tamura et al., 2023^a^ | .24*** | .24*** | — | .23*** | n.s. | — | NR | M | Mediation Analysis |
| van der Putten et al., 2025 (Time 1 camouflaging predicting time 1 mental health constructs) | .16*** | .10*** | — | — | — | — | NR | C; B | Multilevel regression |
| van der Putten et al., 2025 (Time 1 mental health constructs predicting time 1 camouflaging) | .54*** | 86*** | — | — | — | — | NR | C; B | Multilevel regression |
| van der Putten et al., 2025 (Time 1 camouflaging predicting change in mental health constructs over time) | n.s. | n.s. | — | — | — | — | NR | C; B | Multilevel regression |
| van der Putten et al., 2025 (Time 1 mental health constructs predicting change in camouflaging over time) | n.s. | n.s. | — | — | — | — | NR | C; B | Multilevel regression |
| White et al., 2024 | n.s.^a^ | .17** | .24** | — | — | — | NR | N | Hierarchical regression |

**Table S8**

*ASD Camouflaging Subscale Regressions* (NR  = Not Reported; N = no adjustment or covariates noted; B = Bonferroni corrections performed; C = covariates or controlled variables present; M = direct effect in a mediation analysis. * <.05, ** <.01, *** <.001. ^a^ Supplemental materials without *p*-value adjustments.)

|  | Assimilation | Compensation | Masking | Sample Size | Adjusted/Controlled |  |
| --- | --- | --- | --- | --- | --- | --- |
| Moore et al., 2023 | .33*** | n.s. | n.s. | 449 | M | Depression |
|  | .26* | n.s. | n.s. | 449 | M | Anxiety |
|  | 0.41*** | -.12* | n.s. | 444 | M | Mental Wellbeing |
| Pyszkowska, 2024 | 0.29* | — | — | 193 | C | Social Anxiety |
| van der Putten et al., 2025 (Time 1 camouflaging subscales predicting time 1 mental health constructs) | .41*** | — | — | NR | C; B | Depression |
|  | .25*** | — | — | NR | C; B | Anxiety |
| van der Putten et al., 2025 (Time 1 mental health constructs predicting time 1 camouflaging subscales) | .25*** | — | — | NR | C; B | Depression |
|  | .41*** | — | — | NR | C; B | Anxiety |
| van der Putten et al., 2025 (Time 1 camouflaging subscales predicting change in mental health constructs over time) | n.s. | n.s.^a^ | n.s.^a^ | NR | C; B | Depression |
|  | n.s. | n.s.^a^ | n.s.^a^ | NR | C; B | Anxiety |
| van der Putten et al., 2025 (Time 1 mental health constructs predicting change in camouflaging subscales over time) | n.s. | n.s.^a^ | n.s.^a^ | NR | C; B | Depression |
|  | n.s. | n.s.^a^ | n.s.^a^ | NR | C; B | Anxiety |
